# Cryo-electron microscope image denoising based on the geodesic distance

**DOI:** 10.1186/s12900-018-0094-3

**Published:** 2018-12-17

**Authors:** Jianquan Ouyang, Zezhi Liang, Chunyu Chen, Zhuosong Fu, Yue Zhang, Hongrong Liu

**Affiliations:** 1 Key Laboratory of Intelligent Computing and Information Processing, Ministry of Education, College of Information Engineering, Xiangtan University, Xiangtan, 411105 China; 2 College of Physics and Information Science, Hunan Normal University, Changsha, 410081 Hunan China

**Keywords:** Electron cryomicroscopy, Geodesic distance, Similar block, Image denoising, Particle picking, Manifold learning

## Abstract

**Background:**

To perform a three-dimensional (3-D) reconstruction of electron cryomicroscopy (cryo-EM) images of viruses, it is necessary to determine the similarity of image blocks of the two-dimensional (2-D) projections of the virus. The projections containing high resolution information are typically very noisy. Instead of the traditional Euler metric, this paper proposes a new method, based on the geodesic metric, to measure the similarity of blocks.

**Results:**

Our method is a 2-D image denoising approach. A data set of 2243 cytoplasmic polyhedrosis virus (CPV) capsid particle images in different orientations was used to test the proposed method. Relative to Block-matching and three-dimensional filtering (BM3D), Stein’s unbiased risk estimator (SURE), Bayes shrink and K-means singular value decomposition (K-SVD), the experimental results show that the proposed method can achieve a peak signal-to-noise ratio (PSNR) of 45.65. The method can remove the noise from the cryo-EM image and improve the accuracy of particle picking.

**Conclusions:**

The main contribution of the proposed model is to apply the geodesic distance to measure the similarity of image blocks. We conclude that manifold learning methods can effectively eliminate the noise of the cryo-EM image and improve the accuracy of particle picking.

## Background

The theory of three-dimensional (3-D) reconstruction of electron cryomicroscopy (cryo-EM) was defined in the 1960s when Aron Klug and his research group reconstructed the lower-solution 3-D structure of a biological macromolecule by means of Transmission Electron Microscopy (TEM) [1]. Aaron Klug won the Nobel Prize for Chemistry in 1982 for his groundbreaking work. The use of cryo-EM images is considered to be the most efficient method for obtaining a 3-D density map of a complex biological structure [2]. A cryo-EM image is a projection of a biological sample taken by the electron microscope. Environmental factors typically produce noise, and Gaussian noise is generated in the process of digitalizing images. Denoising can eliminate cryo-EM image noise originating from the processes of collection, transmission, and storage of images [3] and can also improve the signal-to-noise ratio of cryo-EM images and the quality of single particles. The high-quality particle picking are used for 3-D reconstruction to obtain the 3-D structure of the biological samples in real space. Therefore, cryo-EM image denoising has great significance for 3-D reconstruction [4, 5].

The noise in cryo-EM images will affects the procedure of adjustment and single particle extraction in 3-D reconstruction [4]. In image processing, since most of the noise comes from interference from electronic devices and the like, the Gaussian and Poisson noise models are often used in actual modeling. At present, most of the denoising algorithms are designed to process white Gaussian noise in images. The probability density function has a normal distribution, and the power spectral density function is a constant [5].

In recent years, with the development of structural biology, cryo-EM images have become increasingly more important. A large amount of multidimensional data generated by standard biomedical imaging modalities, such as electron microscope images and denoising nuclear magnetic resonance image processing, has been used to analyze the distribution of noise in cryo-EM images [6, 7], explore methods for electron microscope image denoising, to reduce noise in images and provide high-quality images for subsequent image processing.

The image block matching algorithm, which is based on the redundancy and correlation of the image information, finds the reference block class to which the candidate block belongs by calculating the distance between the candidate block set X and the reference block set R [8]. The image is segmented according to the noise level in the image [9]. Because images in the image block can be used as a reference block several image blocks are randomly selected as reference blocks. For each image block, the similarity to the reference block is calculated separately, which involves calculating the distance from the reference block. Image blocks that are less than a certain threshold distance from the reference block are considered similar blocks and are classified accordingly until all the image blocks find the corresponding similar block group [10].

Noise type, noise intensity, and image block size are the key factors that affect the performance of denoising in the design of the denoising algorithm based on the image block [8]. Therefore, taking into account the effect of noise on the effective information of the image block, the process analyzes the noise sources of the cryo-EM image and sets the image block size according to the noise standard deviation. The primary methods for cryo-EM image denoising are as follows: two-dimensional (2-D) projection image classification, which focuses on denoising in the transform domain but encounters challenges distinguishing single particles and background noises, and the Taneli Mielikäinen method [11], which introduces the radon transform for image denoising of the single particles but relies on the accurate determination of the common-line of single-particle projection and which cannot extract a single particle from the original cryo-EM image successfully due to the noise. WANG [12] proposed to combine the Zernike matrix and nonlocal means for cryo-EM image denoising, but this method is not suitable for biological macromolecules with nonicosahedral symmetry structure. A nonparametric denoising method combines the contourlet transform and Bayesian estimation, but this method does not take into account the structural characteristics of identical particles in the cryo-EM image.

The denoising method above is based essentially on a Euler metric. Prompted by the increasingly wide use of manifold learning, we introduce the geodesic metric to improve the picture quality effectively. Related research has shown that the structure feature of image blocks can be used to improve the performance of image processing [9]. However, as mentioned, cryo-EM image denoising methods are not integrated into the structure of the image block, so there is still some room for improvement to denoise images [8, 13, 14]. The present paper presents a new method to denoise cryo-EM images by using image blocks. First, blocks that are similar to reference blocks are searched for in the entire image through nonlocal self-similarity prior learning and a similar block-based matching algorithm based on geodesic distance. Second, images are processed with additional Gaussian noise through prior learning; then, image blocks with sparse representation and nonlocal means are denoised. Finally, all the denoised images are reconstructed to obtain the denoised cryo-EM image.

## Results

### Experiment configuration

The image block, noise type, noise intensity, and image block size are the key factors that affect the performance of denoising in the associated algorithm design. Therefore, taking into account the effect of noise on the effective information of the image block, the algorithm analyzes the noise sources of the cryo-EM image and sets the image block size according to the noise standard deviation. Table 1 shows the value of the image block size p and the noise standard deviation *σ* = 50.

**Table 1:** Value of the image block size p and the noise standard deviation *σ* = 50

p	*σ*
6	0 < *σ* < 20
7	20 < *σ* < 30
8	30 < *σ* < 50
9	50 < *σ* < 100

**Fig. 1:**
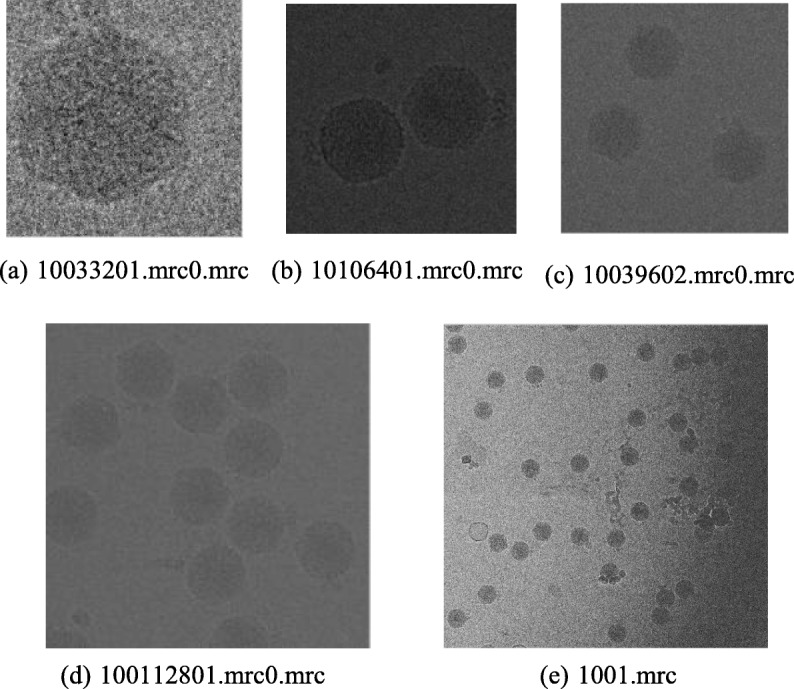
Size of the images of five data sets, ranging from 320 × 320 to 4096 × 4096. With the increase of the size of images, the sizes of the larger images are 4, 9, 16 and 163.4 times that of the original

We used real cryo-EM image data to test our method. This data set contains 2243 images of CPV in total. Particles were randomly selected from the data set and divided into five groups, whose number was different in each group. The size of the images of the five data groups ranged from 320 × 320 to 4096 × 4096. The increasing image size scaled as 4, 9, 16 and 163.4 times that of the original. The specific parameters are shown in Table 2. (Fig. 1).

**Table 2:** Parameter settings of real cryo-EM image data

Parameters	Fig. 1. (a)	Fig. 1. (b)	Fig. 1. (c)	Fig. 1. (d)	Fig. 1. (e)
Image Name	10,033,201.mr c0.mrc	10,106,401.mr c0.mrc	10,039,602.mrc 0.mrc	100,112,801.mrc 0.mrc	1001.mrc
Image Size	320 × 320	640 × 640	960 × 960	1280 × 1280	4096 × 4096
Noise Power	*σ* = 10,20,...,100				
Block size	4 × 4	6 × 6	8 × 8	8 × 8	12 × 12
Dictionary size	64 × 25	128 × 50	256 × 100	256 × 100	576 × 144
Training iterations	20	50	100	200	300
Number of training blocks	6400	12,000	18,000	24,000	117,000

### Experiments on similar block selection

**Fig. 2:**
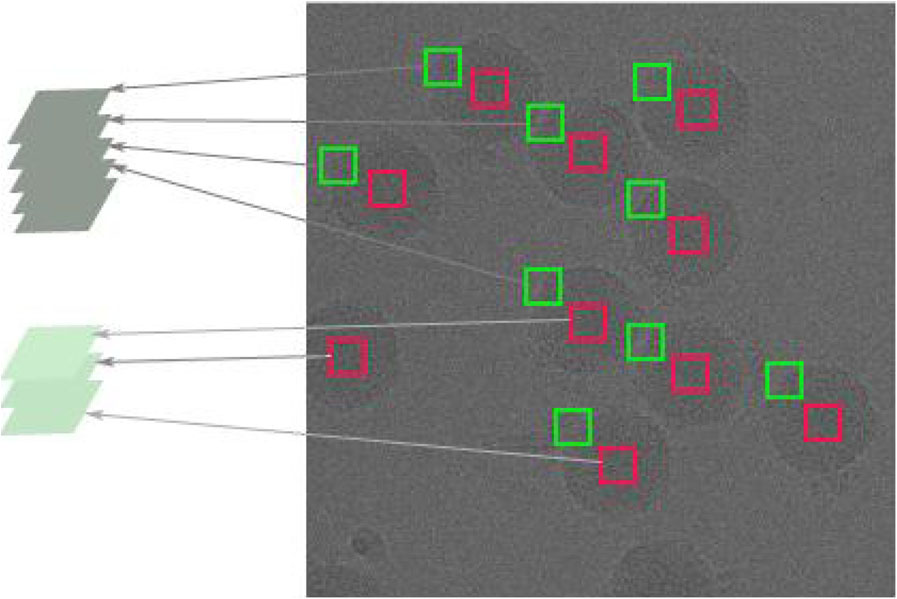
Similar block diagram of the cryo-EM image

Two kinds of methods are widely used to extract image blocks. One is nonrepetitive block extraction using the ‘blkproc’ function in MATLAB, and another is overlapping extraction, which allows the existence of repeated pixels in image blocks [6]. To improve the denoising effect, overlapping extraction is typically used in actual image processing. As shown in Fig. 2, this technique is similar to that of the partial block in Fig. 1(d).

The green box represents similar blocks located on the edge of the particle, and the red box indicates similar blocks whose middle point is at the center of the particle. Because the identities of the CPV virus particles used in the experiment are known, we ignore the noise and other factors; the single particles in the cryo-EM image have similar structure information.

### Experiments on different image block matching algorithms for Cryo-EM images

#### Results and analysis of Cryo-EM image Denoising based on the geodesic distance

For four cryo-EM images with different sizes using the Euclidean distance and geodesic distance to select similar blocks, we demonstrate that the proposed similar block matching algorithm based on the geodesic distance is efficient, and we analyze the influence of the image size and the number of blocks on denoising. For denoising of four cryo-EM images, through similar block matching, we use the Euclidean distance and the measured distance to measure the similarity blocks separately and then recorded the PSNR and SSIM value and the time required for denoising. The experimental parameters are set to *p* = 8 *and σ* = 50, and the experimental results are shown in Table 3.

**Table 3:** Comparison of the experimental results of SSIM and PSNR

Image Name	PSNR of Geodesic distance	PSNR of Euclidean distance	SSIM of Geodesic distance	SSIM of Euclidean distance
Fig. 1(a)	44.92	42.46	0.92	0.90
Fig. 1(b)	44.94	42.47	0.95	0.90
Fig. 1(c)	45.38	43.17	0.89	0.79
Fig. 1(d)	45.65	43.10	0.96	0.89

As the size of the image increases, the PSNR and SSIM values are improved after denoising because when the image block size is fixed, the larger the image, the greater is the number of similar blocks that can be used to learn, and the better the Gaussian component obtained by prior learning can describe the structural features of the image block. For the same image, higher PSNR values and SSIM values were achieved when using the geodesic distance to measure the similarity between the image blocks. Which indicates that accuracy of similar blocks is improved by using similar-block-based matching to enhance the effect of denoising.

Table 4 shows that as the image size scales by 4 times, 9 times, and 16 times, the original denoising time scales by the same amounts. Thus, as the image size becomes larger, the time consumption increases. For the same image, the denoising time when using the geodesic distance is slightly longer than that using the Euclidean distance because the Euclidean distance considers only the gray value of the image. However, the proposed geodesic distance takes into accounts both the gray value and gradient values of the image. As before, the geodesic distance is more accurate (albeit while increasing the denoising time), and a comparison of PSNR and SSIM shows that using the geodesic distance can effectively improve these metrics.

**Table 4:** Comparison of the denoising time

Image Name	Geodesic Distance	Euclidean Distance
Fig. 1(a)	23.70s	21.83 s
Fig. 1(b)	99.97 s	94.58 s
Fig. 1(c)	223.89 s	218.33 s
Fig. 1(d)	417.68 s	401 s

In this paper, the geodesic distance is used to replace the Euclidean distance to measure the similarity between image blocks. At the same time, the computation time increases with increasing accuracy. Relative to other methods, when the noise standard deviation is the same, the proposed method can achieve a higher PSNR value. In addition, the 3-D reconstruction of single particle cryo-EM image is a computationally intensive task; in the complete data processing of obtaining biological macromolecules, denoising represents only a small proportion of data processing. The denoising of cryo-EM images effectively improves the image quality and is helpful to obtain the high-resolution 3-D structure of biological macromolecules.

#### Experiments on different noise standard deviations for Cryo-EM images

**Fig. 3:**
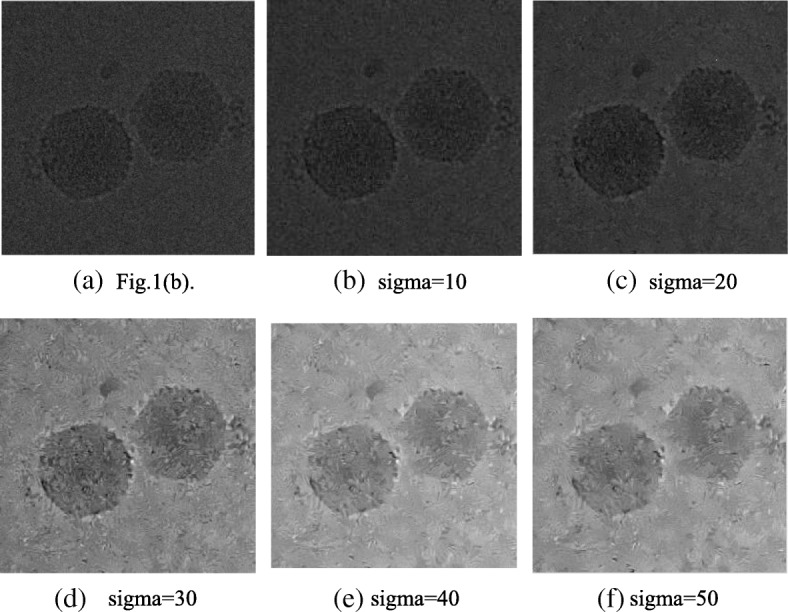
**a** Image of Fig. 1(**b**). **b**, **c**, **d**, **e**, and **f** represent the results of denoising by different noise standard deviations

The proposed method was applied to the denoising of the image shown in Figs. 1(b), and 3(b)-(f) shows the experimental results under different noise standard deviations. The image size is 640 × 640, and the experimental parameters are set as shown in Table 1.

When the electron microscope produces real imaging data, noise is generated during the process; however, as shown in Fig. 3(c), the effect of real data denoising is very effective. With increasing noise in the image, the visual effect of the image is reduced after denoising. Table 5 shows the specific PSNR value and denoising time, where *ΔPSNR* is the difference between the final PSNR value and the initial PSNR value.

**Table 5:** PSNR and denoising time comparison

Noise standard deviation		Initial PSNR	Final PSNR	∆*PSNR*	Denoising time
*σ*	10	28.13	48.93	20.80	80.3
	20	22.11	48.77	26.66	80.28
	30	18.59	46.92	28.33	85.85
	40	16.09	46.31	30.22	99.53
	50	14.15	44.94	30.79	100.28

As shown in Table 5, with the noise standard differential increases, the initial image PSNR value begins to decrease, the PSNR of the denoised image declines gradually, and *ΔPSNR* values increases significantly, which indicates that the proposed method can effectively remove the noise. Furthermore, with increasing noise in the image, the denoising time becomes longer, which indicates that the noise intensity is related to the denoising time.

#### Comparative experimental analysis

To reconstruct a high-resolution 3-D model, cryo-EM images with a low signal-to-noise ratio (SNR) and a complex particle structure must be processed effectively. In this aspect of image processing, the block denoising method represented by BM3D can effectively aggregate similar blocks into a 3-D array and implement co-filtering in the transform domain, which has a favorable effect on image denoising. Cryo-EM images can be regarded as gray images, which can be processed by the BM3D [15], SURE [16], Bayes shrink [17] and K-SVD [18, 19] methods.

- BM3D [15]: This method is a denoising method based on image blocks; it aggregates the similar blocks into a 3-D array and executes collaborative filtering in the transform domain.
- SURE [16]: Stein’s unbiased risk estimate transforms the denoising process into solving the linear equations in the wavelet domain by minimizing the MSE, and the solutions can be used to denoise the image.
- Bayes shrink [17]: The image is processed with the wavelet transform, and the threshold value of each sub-band is self-adaptively solved by Bayes estimation. The wavelet coefficients are transformed using a soft threshold function; finally, the noise is removed.
- K-SVD [18, 19]: This method is an effective and complete method of training sparse signal representation that can achieve image denoising based on a dictionary.

The proposed method is first compared to BM3D, SURE, Bayes shrink and K-SVD, and then all five methods are used to denoise a cryo-EM image to verify the superiority of the method proposed in this paper. In the experiment, the parameters are set to $p=8\kern0.5em and\kern0.5em \sigma =30$.

**Fig. 4:**
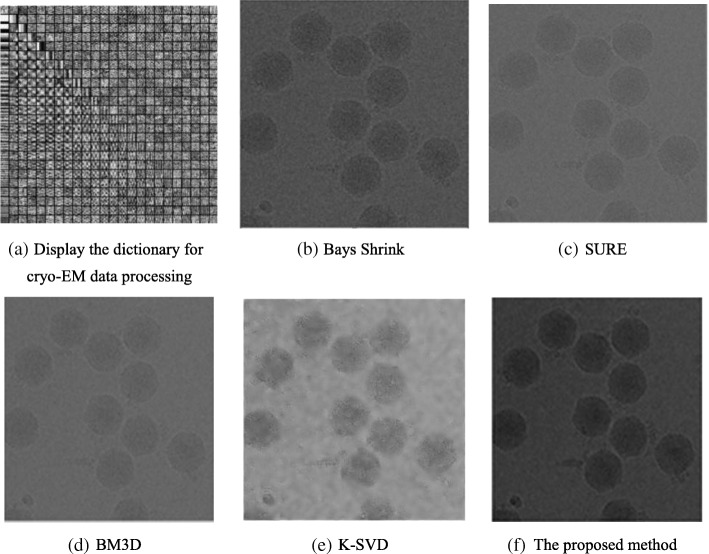
Denoising results under different methods. **a** Trained dictionary of 100,112,801.mrc0.mrc (Fig. 1)d with a block size of 8 × 8. As a contrast experiment, **b**, **c**, **d**, **e**, and (**f**) represent the results of denoising by Bayes shrink, SURE, BM3D, K-SVD, and the proposed method, respectively

Figure 4 shows that the use of the proposed denoising method results in better visual effects. The method can change the original cryo-EM image limitations with low contrast and single particles with a clear outline and edge. The method also facilitates the designs of automatic particle selection algorithms [5], the accuracy of single selected particles, and the resolution of 3-D reconstruction. Moreover, it reduces the total time of 3-D reconstruction.

Table 6 indicates that the proposed method exhibits the highest PSNR value; this result arises because the proposed method takes full advantage of the structural features of the image blocks. The image block averaging can effectively suppress the noise, and through the prior learning of structural information of blocks and sparse presentation, the method denoises image blocks while accurately preserving the details of the image. With increasing noise in the image, the PSNR value is decreased, which indicates that the noise intensity affects the denoising time.

**Table 6:** Comparison of the experimental results of PSNR with different denoising methods

Denoising Method	100,112,801.mrc0.mrc (Fig.4 d)				
	*σ*				
	10	20	30	40	50
BM3D	35.28	33.82	33.17	32.69	32.55
SURE	35.51	34.0	33.43	33.13	32.93
BayesShrink	35.05	33.70	32.99	32.35	31.67
K-SVD	30.89	26.28	24.21	23.13	22.54
The Proposed	49.44	48.52	47.65	45.94	45.65

#### Testing the method on an existing publicly available benchmark data set

To test the validity of the proposed method, public data sets—which are available on the 3-D Electron Microscopy Benchmark website—are used for experimental evaluation. In the experiment, we selected the Ad2 ts 1 Data Set for comparison, in which the parameters were set to $p=8\kern0.5em and\kern0.5em \sigma =40$, and the graphs made in Ad2_ts1_Data Set_I_Test were used.

**Fig. 5:**
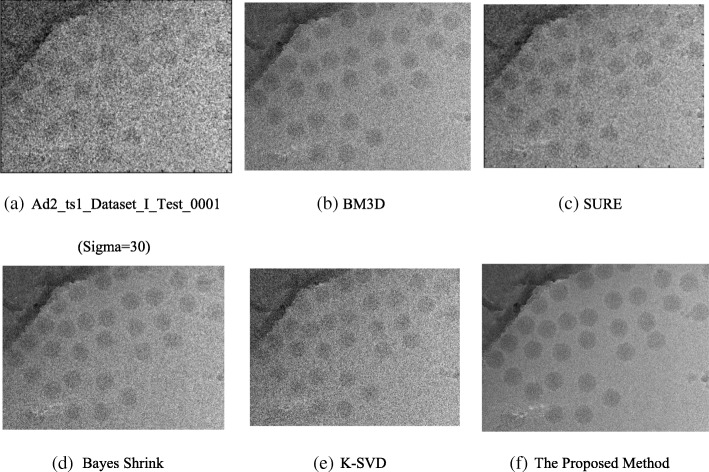
Testing the method on existing publicly available benchmark datasets. Denoising results with different methods. **a** shows the Ad2 ts1 dataset. As a contrast experiment, **b**, **c**, **d**, **e**, and **f** represent the results of denoising by Bayes shrink, SURE, BM3D, K-SVD, and the proposed method, respectively

The experimental results are shown in Fig. 5. For the different methods, the experimental results of the PSNR and time consumed are displayed in Tables 7 and 8, respectively. Figure 5 shows that the proposed denoising method results in better visual effects.

**Table 7:** Comparison of the experimental results of existing publicly available benchmark datasets

Denoising Method	Ad2_ts1_Dataset_I_Test_0001							
	*σ*							
	10	20	30	40	50	60	70	80
BM3D	29.47	26.32	23.49	20.92	20.19	19.58	19.22	18.99
SURE	29.18	24.65	22.51	21.25	20.42	19.64	19.04	18.32
Bayes Shrink	28.24	25.35	23.72	21.29	19.89	18.79	17.42	16.46
K-SVD	18.93	17.68	15.82	15.46	14.89	14.41	13.12	12.97
The Proposed Method	30.87	28.48	25.74	24.56	22.89	21.56	20.65	19.83

**Table 8:** Comparison of experimental results of time with an existing publicly available benchmark datasets

Denoising Method	Ad2_ts1_Dataset_I_Test_0001.tif							
	*σ*							
	10	20	30	40	50	60	70	80
BM3D	389.85	406.79	458.79	491.20	578.46	679.80	625.74	702.20
SURE	226.59	162.80	155.72	382.27	264.26	189.78	206.47	246.57
Bayes Shrink	150.46	168.43	169.78	193.48	209.94	217.23	228.78	249.47
K-SVD	205.79	237.94	178.59	484.55	384.69	445.23	368.75	376.54
The Proposed Method	278.56	204.98	246.49	384.15	496.31	581.32	479.23	379.23

### Experimental results and analysis of 3-D reconstruction

#### Cryo-EM image Denoising

**Fig. 6:**
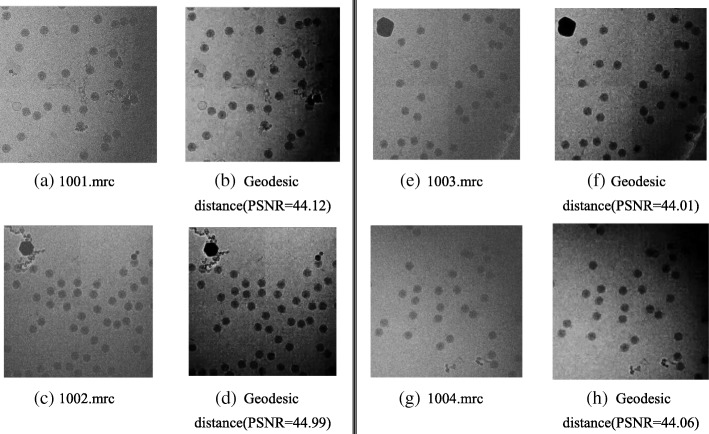
**a**, **c**, **e**, **g** Projections from different directions of the CPV virus with the size of 4096 × 4096. In the experiment, the noise standard deviation is 50. These results verify the validity of the proposed denoising method. The four images are denoised by the proposed method, and the results are shown in (**b**), (**d**), (**f**), and (**h**)

According to the method described in the paper, the denoising experiment is carried out on 7000 CPV capsid particle images of 4096 × 4096 in size on multiple servers; the parameters are set to $p=12\kern0.5em and\kern0.5em \sigma =10$. The partial results after the denoising experiment are shown in Fig. 6. When the noise standard is different, the experimental results of PSNR and SSIM are shown in Table 9.

**Table 9:** PSNR and SSIM values with different noise levels for images (a), (c), (e), and (g) in Fig. 6

Image name	PSNR								SSIM							
	*σ*								*σ*							
	10	20	30	40	50	60	70	80	10	20	30	40	50	60	70	80
1001.mrc	48.61	47.01	46.79	45.54	44.12	42.79	41.28	40.86	0.95	0.94	0.93	0.92	0.90	0.86	0.84	0.80
1002.mrc	48.19	47.23	46.82	45.58	44.99	42.71	41.20	40.49	0.94	0.94	0.93	0.92	0.90	0.86	0.85	0.80
1003.mrc	48.51	47.02	46.56	45.01	44.01	42.99	41.06	40.93	0.95	0.94	0.93	0.91	0.90	0.87	0.86	0.79
1004.mrc	48.42	47.16	46.63	45.81	44.06	42.88	41.14	40.95	0.96	0.95	0.94	0.92	0.90	0.86	0.84	0.80

Table 9 represents the experimental data showing the PSNR and SSIM of the four images in Fig. 6 on different noise levels. The PSNR and SSIM decrease dramatically, and the noise increases and denoising effects worsen as the noise standard deviation increases, which verifies that the noise level strongly affects the performance of the denoising method. For a constant noise standard deviation, the PSNR values of the four images after denoising are similar, as are those of the SSIM, which indicates that the proposed method can remove noise from cryo-EM images effectively.

**Fig. 7:**
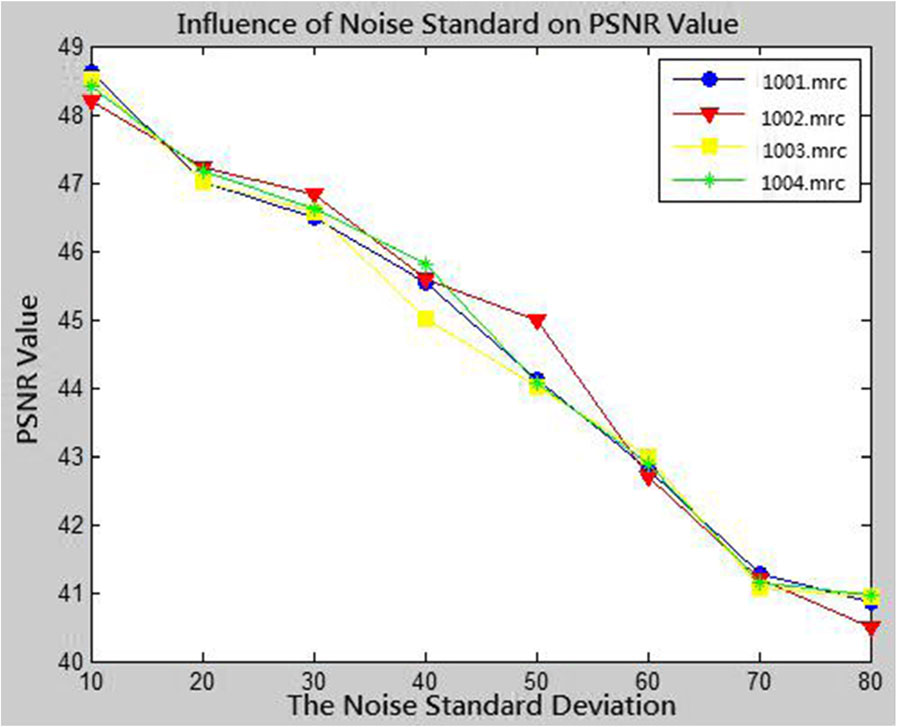
Influences of different noise standard deviations

In Fig. 7, the horizontal coordinates represents the noise standard deviation, and the vertical coordinates represent the PSNR value of the denoised image. With increasing noise standard deviation, the PSNR value decreases significantly. For a constant noise level, the four images have similar PSNR values after denoising, which again verifies that the proposed method can effectively remove noise from cryo-EM images.

#### The results and analysis of the extraction experiment of single particles

The fundamental principle of selecting single particles [5] in their 3-D reconstruction under cryo-EM is that the particles selected must be fine and isolated particles that are unaffected by ice crystals. The use of images with a higher PSNR and contrast obtained after the denoising of the cryo-EM images (as discussed in Cryo-EM image Denoising section) contributes to quick and accurate particle selection. Over the many years of development of electron cryomicroscopy, multiple automatic particle selection algorithms have been proposed. EMAN [20] is an example of an automatic particle selection algorithm. However, due to the limitation of these algorithms and larger deviation of automatic selection, automatic selection is inefficient. However, manual particle selection can achieve satisfactorily accurate performance only when the number of particles is small. With an increasing number of particles, the large consumption of labor becomes prohibitive, and omission and other mistakes can easily occur because of the complex distribution of particles when manual particle selection is selected. Therefore, the false positive rate (FPR) and true negative rate (TNR) are used to judge the quality of single-particle selection, the true positive rate (TPR) is adopted to indicate whether the selection algorithms can accurately recognize all particles, and the time consumed during selection is used to show the time complexity of the selective methods. Table 10 displays the results of the selection of single particles on a CPV virus image by using different methods.

**Table 10:** Comparison of various particle-selection methods

Methods	FPR	TNR	TPR	Time Consumed
Manual selection	0.018	0.005	0.967	150 s
EMAN automatic Selection	0.082	0.204	0.714	28 s
Semi automatic selection	0.040	0.034	0.926	82 s

**Fig. 8:**
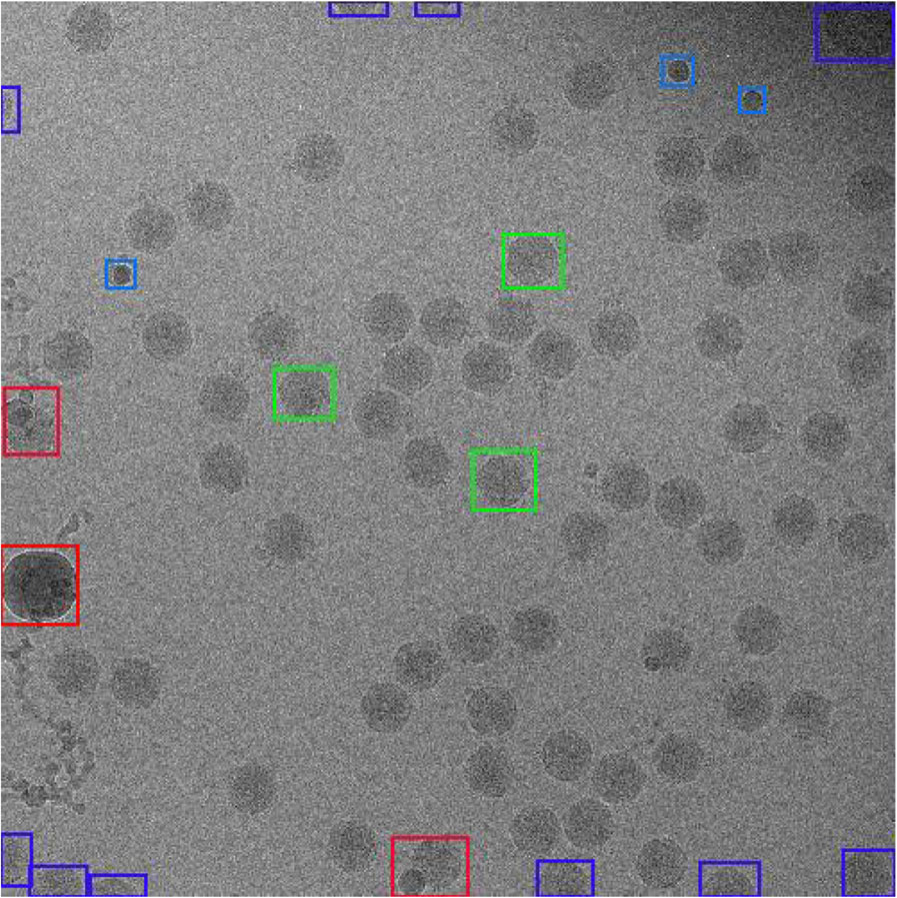
Projection in the cryo-EM image of the CPV virus used for particle selection

The TPR of the manual selection is the highest, but this method also consumes the highest time. In the actual application, the images are automatically selected at first, and then partial particles are manually adjusted when the number of particles is large; thus, more particles can be recognized to the largest extent in less time. Figure 8 shows a projection image of the CPV virus used for particle selection.

**Fig. 9:**
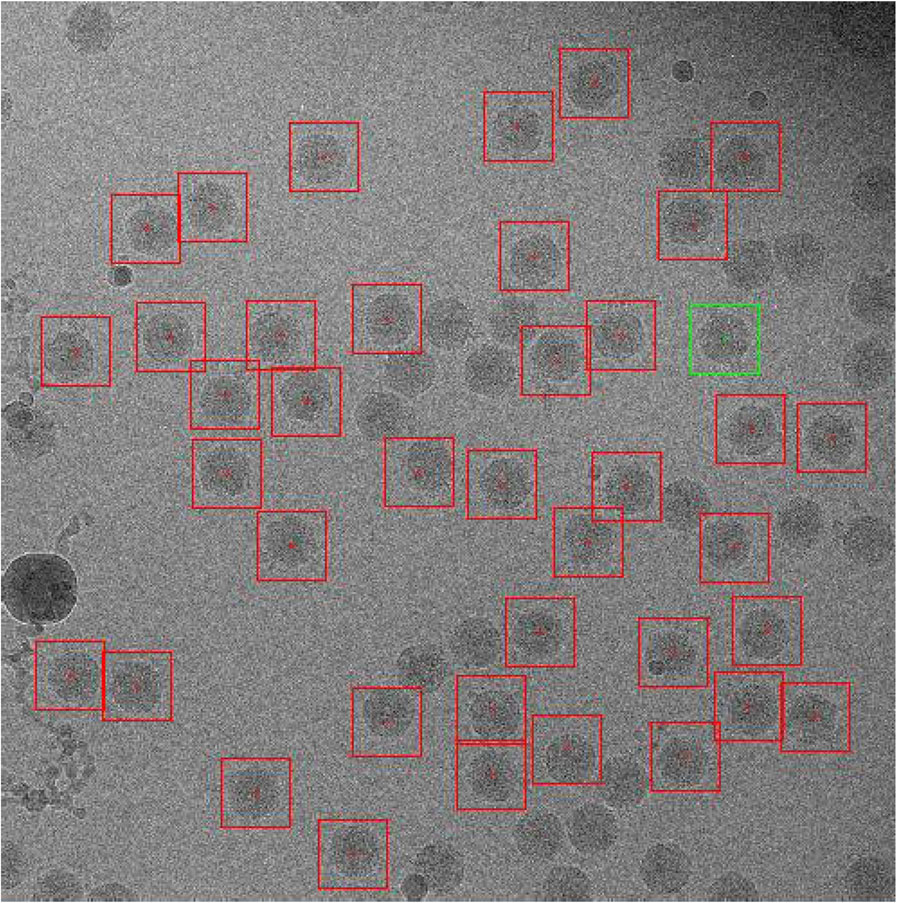
Schematic diagram of extracting single particles of the cryo-EM image

Some particles at the edges of the image are incomplete, some particles in the purple frames are affected by ice crystals, impurities are mixed with particles in the red frame while freezing samples, and the blue frames in the image indicate that these particles would influence the final results of reconstruction. These are all sources of large deviations in the experiment of extracting single particles. To increase the accuracy of single-particle extraction, the above mentioned particles are abandoned at the stage of selecting particles, and only homogeneously distributed and isolated particles remarked with green frames (only partial particles are marked) in the image are selected to carry out 3-D reconstruction. Figure 9 shows a schematic diagram of the manual selection of partial particles.

**Fig. 10:**
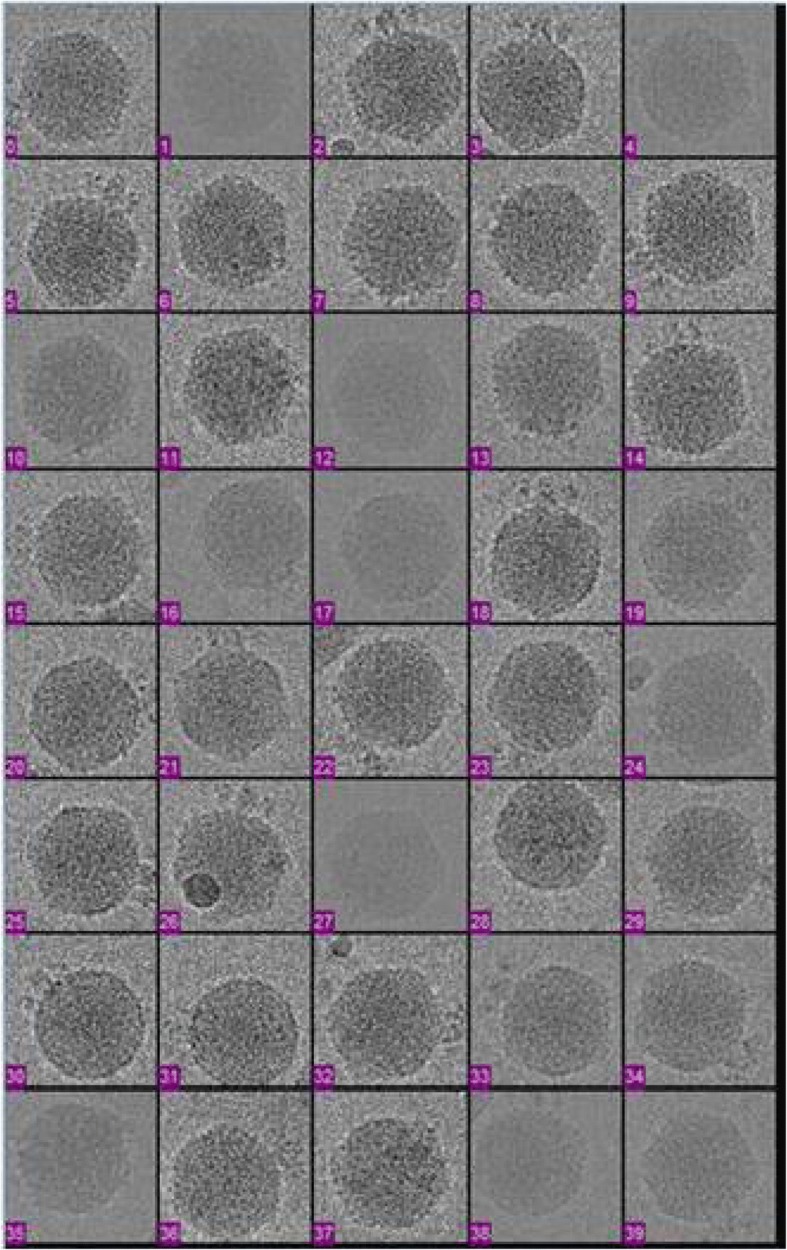
Cryo-EM image of the extraction result of the CPV virus

The distributive rules of mutually selected particles are reviewed. Each single particle can accurately falls into a square frame, in which the selected particles can be stored. Figure 10 shows the schematic diagram of the storage results of 40 single particles through manual selection.

**Fig. 11:**
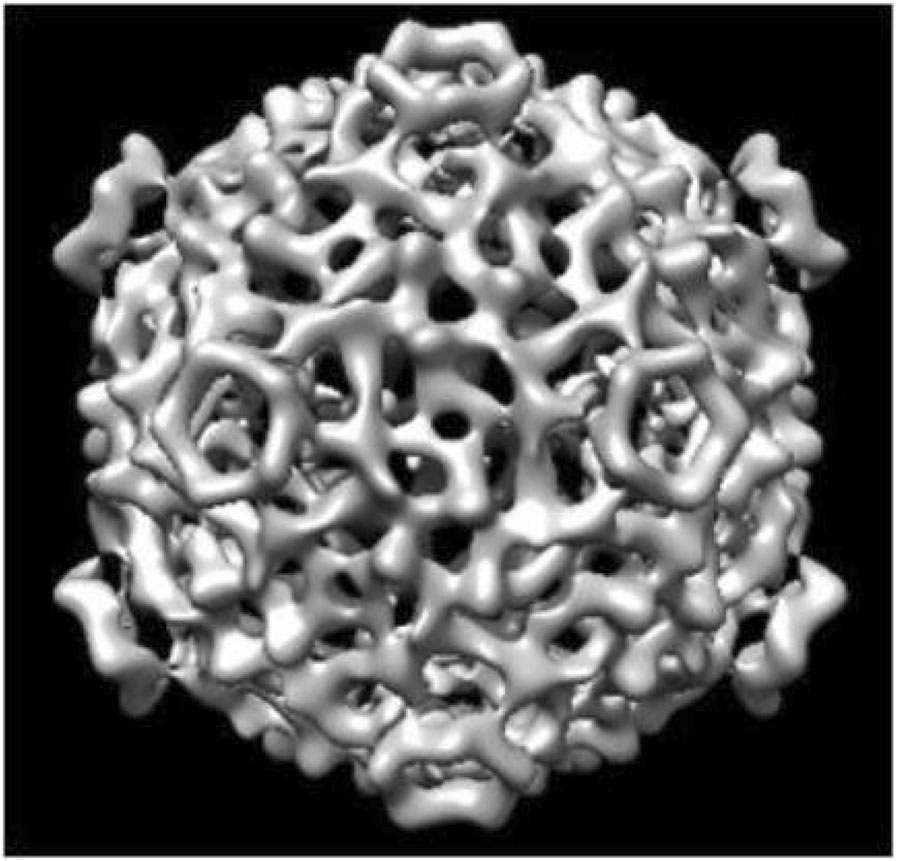
3-D reconstruction of using the central section theorem to obtain the structure of the CPV virus

Parts of single particles in the figure are used in 3-D reconstruction. To obtain the precise structure of the CPV virus, more than thousands of single particles are needed. After the selection of particles, the center and orientation of each particle need to be measured. However, the data of different projections are different and depend on the properties of samples and the resolution requirements of the reconstruction. For the 3-D reconstruction with low resolution, hundreds of icosahedral particles are sufficient. However, 3-D reconstruction with high resolution requires additional particles, and 3-D reconstruction with subnanometer resolution requires thousands of particles. To obtain a structure with near-atomic resolution, approximately 50,000 to 100,000 icosahedral virus particles need to be imaged. In the experiment, a data set of 7000 CPV capsid particle images are used for reconstruction, on which superimposed averaging was performed in the Fourier transform domain. After eliminating parts of particles from which no easily detectable rings could be obtained through the superimposition, 6500 fine particles are used for reconstruction eventually, corresponding to 92.86% of all single particles. The same number of single particles selected from nondenoised cryo-EM images are used to conduct the experiment, from which only approximately 6000 single particles are found that can be used for the reconstruction calculation, accounting for 85.8% of the total number of selected particles, and almost 500 single particles are eliminated, corresponding to a decrease of 7.06%. The extracted single particles are reconstructed by using the central section theorem to obtain the structure of the CPV virus, as shown in Fig. 11. The experimental data show that the extraction accuracy and overall quality of single particles can be improved by first denoising the cryo-EM images and then conducting semiautomatic selection of single particles; in this way, the 3-D reconstruction resolution can be enhanced.

## Discussion

We evaluate the competing methods from four aspects: PSNR, contrast, time and visual quality.

### PSNR

The results are presented in Table 6. We have compared the PSNR of five methods on five noise levels: Δ = 10, 20, 30, 40, and 50. The K-SVD method performs worst; BM3D, SURE and Bayes shrink obtain results similar to those of PSNR, and the method we proposed performs better than PSNR, with an improvement of approximately 14 dB relative to BM3D, SURE and Bayes shrink. These results validate that the proposed method has a significant ability to denoise images.

### Contrast

The proposed method of denoising can significantly improve the PSNR of the image, which is indicated from the visual effect of the image; the contrast of the cryo-EM image is improved, and a better visual effect is obtained because high similarity exists among each single particle in the cryo-EM image used in the experiment. The substitution of the geodesic distance in place of the Euclidean distance improves the accuracy of searching similar blocks when similar blocks are matched and when the denoising effect is implemented through prior learning.

### Time

The method proposed in this paper requires a longer time to denoise the cryo-EM images because the data type of the cryo-EM image is 32-bit floating point numbers; consequently, as the image size increases, the time taken to read the images increases, and the time required to calculate the distance between image blocks with the increase of the numbers of image blocks, which need to be denoised during the matching of image blocks. Additionally, in this paper, the prior learning is first performed on similar blocks, and then the groups of image blocks are denoised; therefore, the denoising time is longer than that of the other four methods.

Table 11 shows that with increasing noise in the image, the denoising time also shows an increasing trend. The time required for SURE and Bayes shrink is relatively low, and the time required for the Bayes shrink is approximately 1.7 times that of SURE. When the noise level is the same, SURE requires a slightly longer denoising time than Bayes shrink. Relative to SURE and Bayes shrink, the BM3D algorithm takes significantly more time. The proposed method takes the longest time, approximately 10 times that of the BM3D algorithm.

**Table 11:** Comparison of the experimental results of time with different denoising methods

Denoising Method	100,112,801.mrc0.mrc (Fig.4 (d))				
	*σ*				
	10	20	30	40	50
BM3D	34.0	33.1	33.2	35.1	36.8
SURE	21.7	21.4	21.5	21.4	21.4
BayesShrink	41.3	38.2	37.6	37.3	37.1
K-SVD	205.21	69.03	36.59	27.60	25.11
The proposed Method	326.27	328.41	345.59	402.54	417.68

Efficiency is another factor to evaluate the methods. We have compared the speed of the 5 methods under the same environment as presented in Table 2. In Table 11, we show the runtime results on the five noise levels Δ = 10, 20, 30, 40, and 50. Considering the application of parallel computing and the high-speed development of computation modules, the runtime makes up a smaller proportion of evaluation than before.

### Visual quality

The visual quality plays an important role in the evaluation of any denoising method because human beings are the ultimate judge of image quality. Figure 4 shows the images denoised by the five method. The image processed by the K-SVD remains fuzzy. In the images processed by SURE and BM3D, the edges cannot be clearly distinguished. Comparing the two images associated with Bayes shrink and the proposed method, we find that higher contrast is achieved by the latter. In general, the proposed method demonstrates a strong ability to denoise images.

## Conclusions

A similar block matching algorithm based on the geodesic distance has been proposed in the paper and applied to the design of a denoising algorithm based on image blocks. The method is based on similar block matching, using the geodesic distance to measure the similarity of image blocks. The method searches for the similar blocks in the whole image field, enhancing the performance of denoising by improving the accuracy of similar blocks and denoising each group of similar blocks separately.

Similar blocks with additional Gaussian noise were treated by prior learning. Finally, the entire denoised image block was used to reconstruct the denoised cryo-EM image. The experiments show that the proposed method can effectively eliminate noise in the cryo-EM image.

## Methods

### State of the art on image Denoising based on image blocks

Dictionary learning has broad applications, including image recognition, denoising and restoration [18, 19]. The goal of dictionary learning is to find a sparse approximation solution to represent a class of signals under an appropriate measure. Moreover, sparseness can often be used to avoid overtraining. Current dictionary learning algorithms focus on selecting the vector on the Euclidean space. However, data points often modeled by a Riemannian manifold are critical to applications involving image denoising [20, 21].

In the paper, the image is divided into blocks according to the noise level in the image. Any image block in the image domain can be used as a reference block, and some image blocks are randomly selected as the reference blocks. For each candidate image, we calculate the similarity of each block to that of the entire reference block, that is, we calculate the distance between it and the reference block. When the distance is less than a fixed threshold value, we classify these two image blocks as similar and place them in the same similar block group; the process continues until all of the candidate image blocks are placed into a corresponding block group.

### Similar block matching algorithm based on the geodesic distance

In the existing similar block matching algorithm [21], the Euclidean distance between the candidate block S_x_ and the reference block set ${\left\{{\mathrm{S}}_{\mathrm{x}}\right\}}_{\mathrm{k}=1}^{\mathrm{K}}$ is typically used to calculate the similarity. However, the Euclidean distance does not take into account the local connectivity. Moreover, the image block subspace is not entirely Euler space. To overcome this limitation, in the paper, the Euclidean distance is replaced by the geodesic distance [22] to evaluate the similarity of image blocks. The geodesic distance considers the intrinsic influence on the image space, and its computation is not complex.

The image block is composed of pixels. For the two given image blocks, S_A_ and S_B_, their sizes are both p × p; ${\mathrm{d}}_{\mathrm{R}}\left({\mathrm{S}}_{{\mathrm{A}}_{\mathrm{i}}},{\mathrm{S}}_{{\mathrm{B}}_{\mathrm{i}}}\right)$ is used to represent the geodesic distance between the two image blocks in the i-th pixels.

**Fig. 12:**
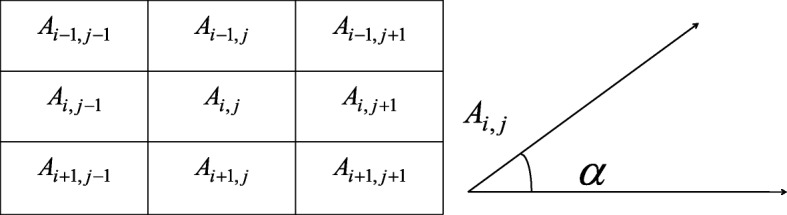
View of an angle; value [Ai] represents the gray values of i-th pixel point in an image block SA. α represents the angle of the i-th point of the image block SA, which is the angle between the direction of the maximum change of the gray value and the minimum direction

Calculate the weight of the i-th pixel point weight1 and weight2:

1 $$ weight1={0.5}^{\ast }{\left( value\left[ Ai\right]- value\left[ Bi\right]\right)}^2, $$

where value [Ai] represents the gray values of the i-th pixel point in an image block *S*_*A*_ and value [i] represents the gray values of the i-th pixel point in image block *S*_*B*_.

2 $$ weight2={0.5}^{\ast}\left( tAi+ tBi+\tan \left|\alpha -\beta \right|\right), $$

where tAi represents the gradient value of the i-th point in the image block *S*_*A*_. tBi represents the gradient value of the i-th point in the image block *S*_*B*_. *α* represents the angle of the i-th point of the image block *S*_*A*_, which is the angle between the direction of the maximum change of the gray value and the minimum direction. *β* represents the angle of the i-th point of the image block *S*_*B*_, which is the angle between the direction of the maximum change of the gray value and the minimum direction. Figure 12 shows a view of an angle.

Here, $\upalpha =0,\frac{\uppi}{4},\frac{\uppi}{2},\frac{3\uppi}{4},\uppi$, and eight pixels exist around pixel A_i, j_. The gray value changes are calculated to find the direction of the maximum change in the guidance of the maximum change and the direction of the smallest change, and the angle is defined as α. In the same way, we know that$\upbeta =0,\frac{\uppi}{4},\frac{\uppi}{2},\frac{3\uppi}{4},\uppi$. Consequently, the geodesic distance between two points Ai and Bi, which are located at the same position in different image blocks, is defined as

3 $$ d\left({S}_{Ai},{S}_{Bi}\right)= weight1+ weight2. $$

Therefore, the geodesic distance between the image blocks S_A_ and S_B_ is

4 $$ \mathrm{d}\left({\mathrm{S}}_{\mathrm{A}},{\mathrm{S}}_{\mathrm{B}}\right)=\frac{1}{{\mathrm{p}}^{\ast}\mathrm{p}}{\sum}_{\mathrm{i}=1}^{\mathrm{p}\ast \mathrm{p}}\mathrm{d}\left({\mathrm{S}}_{{\mathrm{A}}_{\mathrm{i}}},{\mathrm{S}}_{{\mathrm{B}}_{\mathrm{i}}}\right), $$

where i is the i-th pixel point of the image block and i = 1, 2, …, p × p. Comparing d(S_A_, S_B_) with the fixed threshold T, if d(S_A_, S_B_) < T, we define the image blocks A and B as similar blocks; otherwise, the image blocks A and B are not similar. The value of the fixed threshold T is related to the size of the image blocks. The larger the image block, the larger the value of T.

**Fig. 13:**
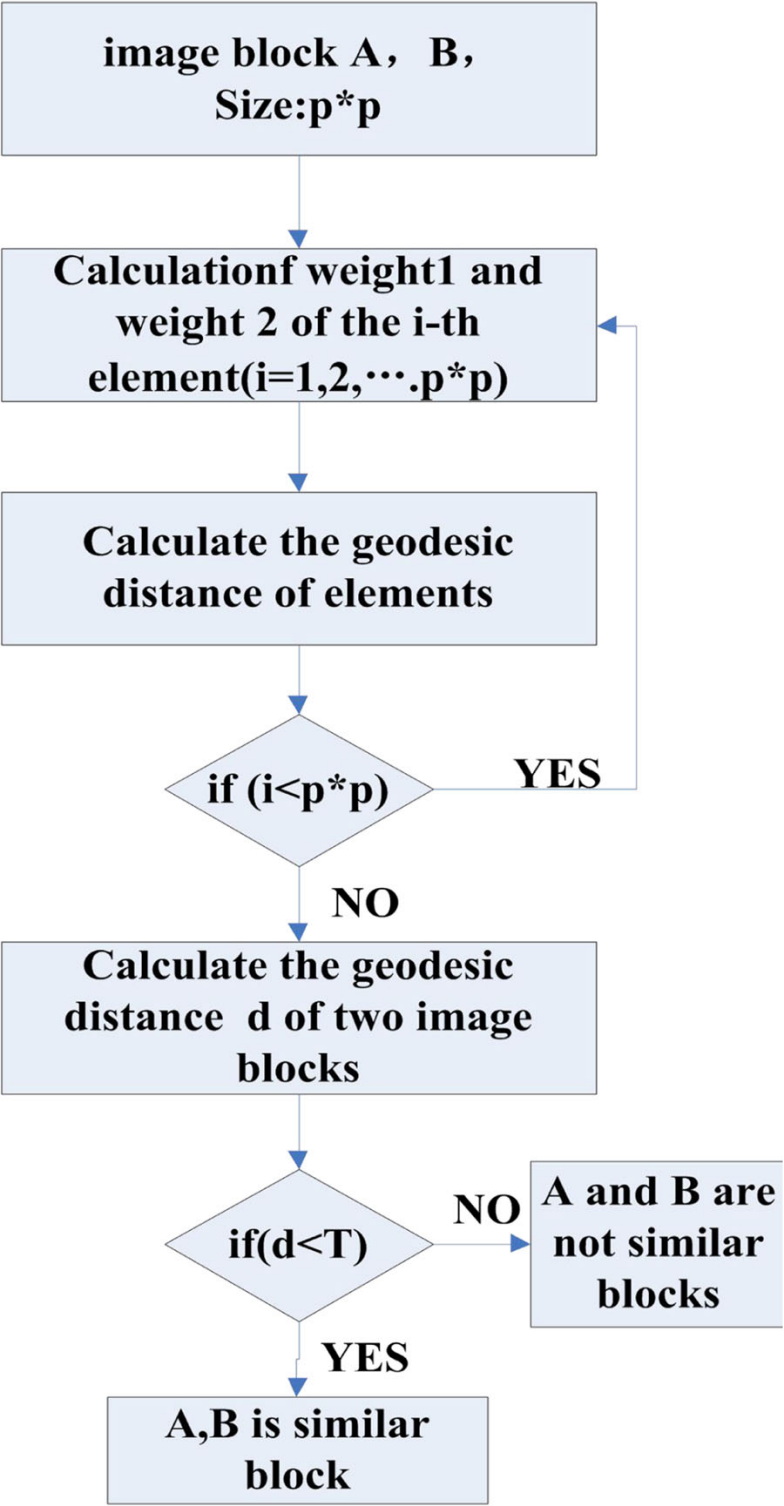
Evaluation flow chart of a similar block

In the image domain, we can use a 2-D discrete function to represent the image, and the gradient direction is the direction of the maximum change of the gray value, so this paper uses the gradient value and the gray value of the image to describe the geodesic distance. When the distance between two image blocks is less than the threshold, the two image blocks are considered to be similar blocks. The value of the threshold is related to the size of the selected image block. Figure 13 shows the flow chart of using the geodesic distance to measure the similarity of the image block.

In the paper, we use the proposed method to evaluate the similarity of the image blocks; then, the geodesic distance is used to select the similar block for the reference blocks. The detailed similar-block-based matching algorithm based on the geodesic distance is shown in Table 12.

**Table 12:** Image block matching algorithm

Alg. 1: Similar block matching algorithm based on geodesic distance	
Input: candidate block *S*_*x*_, reference block set ${\left\{{S}_n\right\}}_{n=1}^N$ Output: *S*_*x*_ ∈ *S*_*n*_	
Step1. According to the Geodesic distance formula, calculate the geodesic distance between A and B. Step2. Compare the value of *d*_*n*_(*n* = 1, 2……*N*), select the minimum value *d*_*n*_. Step3. Determine candidate block *S*_*x*_ which is similar to the reference block *S*_*n*_ Step4. Repeat steps 1–3 until all image blocks are completed.	

The accuracy of similar blocks has a strong impact on the accuracy of the subsequent learning of similar blocks and affects the accuracy of the dictionary and sparse coding of the similar blocks, which could affect the final denoising result. The proposed block matching method is based on the geodesic distance, which enhances the accuracy of the similar block group and lays a foundation for establishing a more precise denoising model.

### Evaluation Indicator of the Denoising effect

A suitable denoising algorithm would remove the noise to the maximum extent, maintaining the integrity of the valid information of the original image while having relatively low computational time complexity. It is typical to evaluate the performance of denoising methods with objective evaluation indexes and visual effects of images.

When evaluating the denoising performance of a specific algorithm, in addition to comparing the visual effect, indicators such as the PSNR, mean square error (MSE), SNR and execution time are used to measure the advantages and disadvantages of the algorithm.$MSE=\frac{1}{N^2}\sum \limits_{i,j=1}^N{\left(\overset{\wedge }{X_{i,j}-{X}_{i,j}}\right)}^2$, where X is the original image with noise and $\hat{X}$ is the estimate of the original image X that is the denoised image.

$PSNR=10\log 10\frac{\max \left({x}^2\right)}{MSE}$, where $SNR=10\log 10\frac{P_s}{P_n}$, *P*_*s*_ denotes the effective signal power, and *P*_*n*_ denotes the noise power.

For image processing, apart from PSNR and MSE used to evaluate an algorithm, the structural similarity index measurement system (SSIM) is also a reliable indicator that is based on the correlation of neighboring pixels in natural images. SSIM avoids tallying up different kinds of errors to depict the image differences before and after denoising. The closer to one the SSIM value between the noise image X and the denoised image $\overset{\Lambda}{X}$ is, the more similar in structure they are.

$SSIM\left(X,\overset{\wedge }{X}\right)=\frac{\left(2{\mu}_1{\mu}_2+{c}_1\right)\left(2{\sigma}_{12}+{c}_2\right)}{\left({\mu}_1^2+{\mu}_2^2+{c}_1\right)\left({\sigma}_1^2+{\sigma}_2^2+{c}_1\right)}$, where *μ*_1_and *μ*_2_ denote the average, ${\sigma}_1^2$ and ${\sigma}_2^2$ denote the variance, *σ*_12_ denotes the covariance, and *c*_1_ and *c*_2_ are constants close to 0. PSNR and MSE are based on the statistical model of the image grayscale value, while SSIM is based on differences of image structures. In practical use, it is typical to combine subjective evaluation and objective evaluation to evaluate an algorithm.

The CTF plays a significant role in the comparison of cryo-EM data. To obtain a high-resolution 3-D reconstruction of a virus by a cryo-EM image, it is necessary to implement a CTF correction for the micrographs. It is hard to distinguish the positions of the CTF zeros accurately due to the low signal-to-noise ratio of the cryo-EM image. To avoid an inaccurate measure of the positions of the CTF zero blurred by attenuation at high frequency, we use a Gaussian curve to compensate for the attenuation of the Fourier transform of the image at high frequency; in this way, the amplitudes of the two CTF zeros at the curve are the same value.

### Proposed Cryo-EM image Denoising

The purpose of cryo-EM image denoising is to remove the noise in the image, improve the contrast and the SNR of the picture, and provide sufficient information for the following single-particle selection and 2-D projection image classification. Our method implements prior learning of the image block and sparse representation and then uses the dictionary representation to denoise the image block. We use the method of learning from similar blocks to obtain the dictionary, which avoids the limitation of using the discrete cosine transform (DCT) dictionary [23, 24]. The dictionary denoising theory is based on the ideal image having sparse representation under the appropriate overcomplete dictionary; the noise can destroy the sparse representation. By choosing or designing appropriate dictionaries, the sparse representation of natural images in the dictionary can be achieved to reduce or eliminate the noise.

A significant number of identical particles exist in the cryo-EM images. The image block can effectively use the characteristics of these identical particles and achieve improved experimental results. In the paper, the similar block matching method based on the geodesic distance is combined with the nonlocal self-similarity (NSS) prior knowledge of image blocks [9] to search the similar blocks of the reference blocks in the whole image domain, followed by the process of prior learning with the image blocks. The proposed method takes into account the distance in the manifold space and uses the geodesic distance to select similar blocks accurately. In addition, the proposed method gives the prior internal knowledge and the external prior knowledge of the similar blocks.

The hypothesis observation image is *y*, the free-noise image is *x*, the noise is *v*, and *y* = *x* + *v*; here, $\mathrm{PSNR}=10\log 10\frac{255^2}{\mathrm{MSE}}$. Thus, the cryo-EM image denoising problem is transformed into obtaining an estimate $\widehat{x}$ of an image *x* by observing the image y so that ${\left\Vert \mathrm{x}{-}_{\mathrm{x}}^{\hat{\mkern6mu}}\right\Vert}^2$ the denoising problem can be minimized. This process determines the minimum MSE, which can be used to obtain the maximum PSNR value and optimal denoising result.

A cryo-EM image was decomposed into image blocks; N reference blocks were chosen. In our process, the geodesic distance is used to select the similar blocks, and then all the similar blocks are clustered into N similar blocks, each of which contains M similar blocks. *y*_*m*_ represents the image block in the picture *y*, and *x*_*m*_ represents the image block in the picture *x*. According to the formula $\mathrm{PSNR}=10\log 10\frac{255^2}{\mathrm{MSE}}$, to make the image PSNR as large as possible, the MSE must be as small as possible. Therefore, the image denoising problem can be converted to the minimum MSE problem, that is,

5 $$ \underset{MSE}{\underbrace{\left\langle {\left|\overset{\Lambda}{x}-x\right|}^2\right\rangle }}=\frac{1}{N^{\ast }M}\sum \limits_{n=1}^N\sum \limits_{m=1}^M\underset{MSE}{\underbrace{\left\langle {\left|\overset{\Lambda}{x_m}-{x}_m\right|}^2\right\rangle }}, $$

**Fig. 14:**
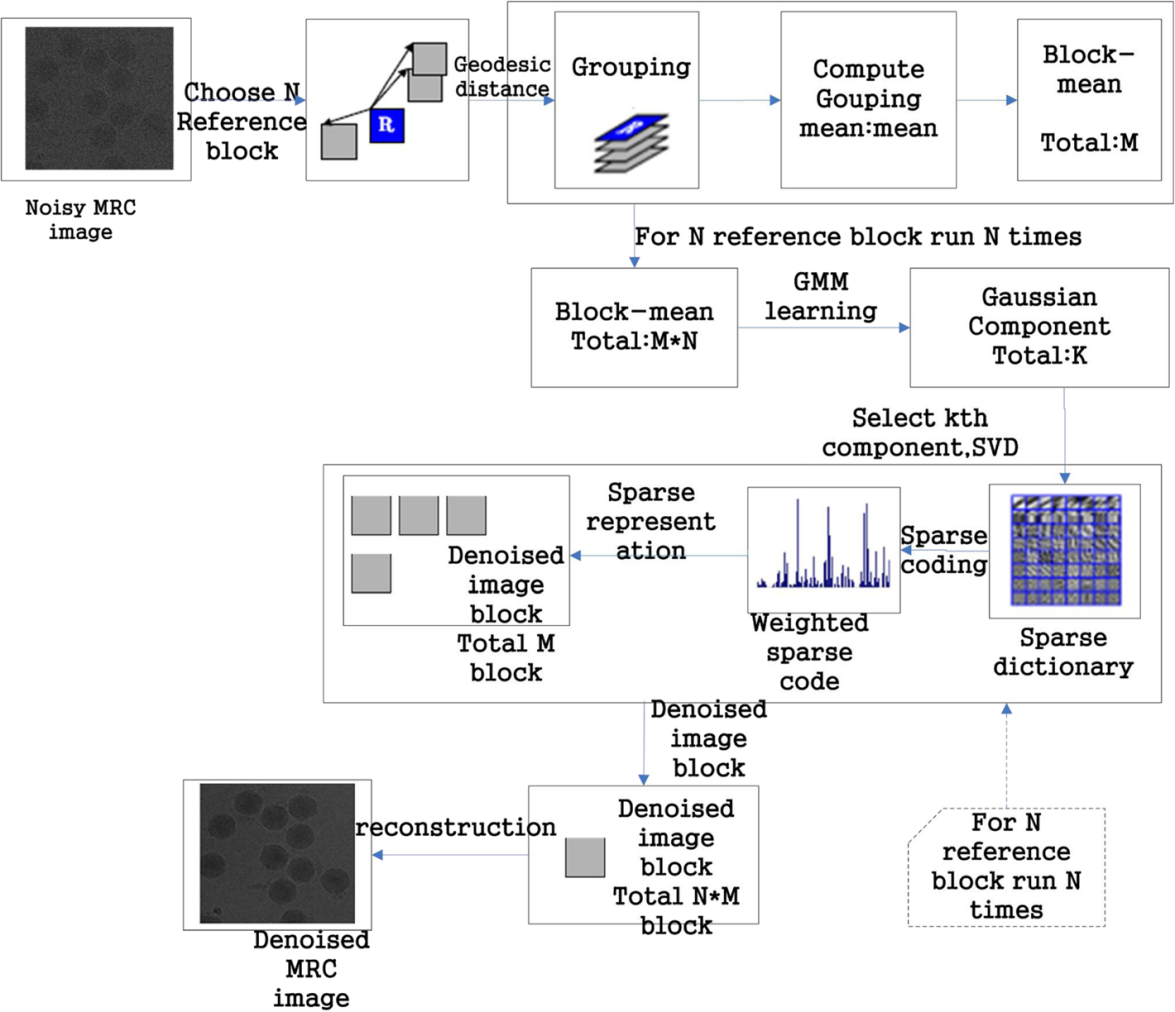
Proposed denoising flowchart

Here, $\left\langle u\right\rangle =\frac{1}{p^{\ast }p}\sum \limits_{i=1}^{p^2}{u}_i$ because the nonlocal means can suppress the noise, and the dictionary can effectively represent the nonnoise signal in the image. Therefore, combined with the nonlocal mean and dictionary representation, we can obtain the denoised image block. By solving the dictionary D and sparse coding coefficient, the purpose of denoising image blocks can be achieved, and the image blocks can ultimately be used to reconstruct the denoised cryo-EM image. The denoising flow chart is shown in Fig. 14.

${\left\{{\mathrm{y}}_{\mathrm{m}}\right\}}_{\mathrm{m}=1}^{\mathrm{M}}$refers to M similar blocks with size p × p in the image y; here, ${\mathrm{y}}_{\mathrm{m}}\in {\mathrm{R}}^{{\mathrm{p}}^{2\ast 1}}$. The mean value of M image blocks is expressed by *μ*_*y*_, where ${\mu}_y=\frac{1}{M}{\sum}_{m=1}^M{y}_m,\overline{y_m}={y}_m-{\mu}_y$, $\overline{Y}\underline {\underline{\Delta}}\left\{\overline{y_m}\right\},m=1,\dots, M$, and $\overline{Y}\underline {\underline{\Delta}}{\left\{\overline{y_{n,m}}\right\}}_{m=1}^M,n=1,2,\dots, N;m=1,\dots, M$.

The next step is to implement a priori learning of $\overline{{\mathrm{Y}}_{\mathrm{n}}}$ to calculate the K Gaussian distribution.

According to the sparse redundancy of images, the probabilistic representation is calculated by$\left\{\overline{{\mathrm{Y}}_{\mathrm{n}}}\right\}:\mathrm{P}\left(\overline{{\mathrm{Y}}_{\mathrm{n}}}\right)={\sum}_{\mathrm{k}=1}^{\mathrm{K}}{\uppi}_{\mathrm{k}}{\prod}_{\mathrm{m}=1}^{\mathrm{M}}\mathrm{N}\left(\overline{{\mathrm{y}}_{\mathrm{n},\mathrm{m}}}|{\upmu}_{\mathrm{k}},{\sum}_{\mathrm{k}}\right)$.

Therefore, the global target likelihood function can be expressed as $\mathrm{L}=\prod \limits_{\mathrm{n}=1}^{\mathrm{N}}\mathrm{P}\left(\overline{{\mathrm{Y}}_{\mathrm{n}}}\right)$ for the convenience of the following calculation, namely, the logarithmic function of the target likelihood

6 $$ \mathrm{lnL}={\sum}_{\mathrm{n}=1}^{\mathrm{N}}\ln \left({\sum}_{\mathrm{k}=1}^{\mathrm{K}}{\uppi}_{\mathrm{k}}{\prod}_{\mathrm{m}=1}^{\mathrm{M}}\mathrm{N}\left(\overline{{\mathrm{y}}_{\mathrm{n},\mathrm{m}}}|{\upmu}_{\mathrm{k}},{\sum}_{\mathrm{k}}\right)\right). $$

Through GMM learning [25], we can obtain the K Gaussian distribution, which can describe the structural characteristic of the image block. In this context, the maximum posterior probability of each block is obtained by using the Bayesian method, and the most suitable Gaussian component for each group of similar blocks is obtained. Then, the dictionary of the similar block group is obtained to denoise the image block. The algorithm is described in Table 13.

**Table 13:** Bayesian approach seeking dictionary

Alg. 2: Use Bayesian method to find the dictionary D of similar block group	
Input: Similar block group ${\overline{Y}}_n,n=1,2,..N$, K Gaussian distribution {N(*μ*_*k*_, ∑_*k*_)}*K* = 1, 2, …, *k* through GMM leaning. Output: Gaussian component of similar block group ${\overline{Y}}_n$ corresponded dictionary D.	
Step1. initialization *n* = 1,k = 1. Step2. Apply the formula $\ln P\left(k\left|\overline{Y}\right.=\right)\sum \limits_{m=1}^M\ln N\left({y}_m\left|\overline{0},{\sum}_k\right.\right)-\ln C$ to calculate $\ln P\left(k\left|\overline{Y}\right.\right)$ when taking the k-th Gaussian component. Step3. Repeat step 2, total of K times for calculating $\ln P\left(k\left|\overline{Y}\right.\right)$ values. Step4. Compare $\ln P\left(k\left|\overline{Y}\right.\right),k=1,2,\dots, k$, get the maximum $\ln P\left(k\left|\overline{Y}\right.\right)$, its corresponding Gaussian distribution can describe similar block group *Y*_*n*_, its covariance matrix is ∑_k_. Step5. For SVD decomposition, get dictionary *D*_*n*_ of similar block group *Y*_*n*_. Step6. Repeat steps 2–5, a total of N times, until the output N is a dictionary D.	

According to the sparse representation of the image, ${\overline{\mathrm{y}}}_{\mathrm{m}}=\mathrm{D}\upalpha +\mathrm{v}$, where v is noise. Dictionary D is known. By solving α, the sparse representation of the image block can be obtained, and the image block’s denoising can be realized. In the paper, the constraint conditions of the sparse coding model are expressed as

7 $$ {{}_{\upalpha}{}^{\min }{\left\Vert {\overline{\mathrm{y}}}_{\mathrm{m}}-\mathrm{D}\upalpha \right\Vert}_2^2+\left\Vert {\mathrm{W}}^{\mathrm{T}}\upalpha \right\Vert}_1, $$

where *α* is the sparse coding coefficient and *w* is the weight of the *α* vector. According to the method provided by PGPD [13, 25],

8 $$ \widehat{\mathrm{a}}=\operatorname{sgn}\left({\mathrm{D}}^{\mathrm{T}}{\overline{\mathrm{y}}}_{\mathrm{m}}\right)\bigodot \max \left(\left|{\mathrm{D}}^{\mathrm{T}}{\overline{\mathrm{y}}}_{\mathrm{m}}\right|-\mathrm{w}/2,0\right), $$

where $$ {\mathrm{w}}_{\mathrm{i}}=\frac{\mathrm{c}\ast 2\sqrt{2}{\upsigma}^2}{\uplambda_{\mathrm{i}}+\upvarepsilon} $$ and $$ {\mathrm{D}}^{\mathrm{T}}{\overline{\mathrm{y}}}_{\mathrm{m}}=\mathrm{z} $$,

which is typically written in the following form:

$\widehat{\mathrm{a}}=\operatorname{sgn}\left({\mathrm{z}}_{\mathrm{i}}\right){\left(\left|{\mathrm{z}}_{\mathrm{i}}\right|-\frac{{\mathrm{w}}_{\mathrm{i}}}{2},0\right)}_{+}$, where (a)_+_ = max(a, 0)and sgn(∙) is a symbolic function. We define a function SoftMAP:

*SoftMAP*(*g*_*i*_, *τ*_*i*_) = sgn(*g*_*i*_)(| *g*_*i*_| −*τ*_*i*_)_+_ is the sparse coding of the similar block group

9 $$ \widehat{\upalpha}=\mathrm{SoftMAP}\left(\mathrm{z},\frac{\mathrm{w}}{2}\right), $$

to minimize the impact of noise in the image blocks and inaccurate similar blocks grouping in Gaussian mixture models (GMMs). The proposed geodesic distance can improve the accuracy of the similar block group. Moreover, this process uses a combination of the dictionary D and weighted sparse coding $\widehat{\upalpha}$ to attain the denoised image block ${\widehat{\mathrm{x}}}_{\mathrm{m}}$:

10 $$ {\widehat{\mathrm{x}}}_{\mathrm{m}}={\upmu}_{\mathrm{y}}+\mathrm{D}\widehat{\upalpha}, $$

First, the estimated value of the image blocks in each similar block group is obtained, and then the denoised image $\widehat{\mathrm{x}}$ is reconstructed by aggregating all the denoised image blocks. When some estimated values appear in a position of the image, the final estimate is obtained by using the weighted average. Through the formula ${\left({\upsigma}^{\left(\mathrm{t}\right)}\right)}^2=\upeta \ast \left({\upsigma}^2-{\left\Vert \mathrm{y}-{\mathrm{y}}^{\left(\mathrm{t}-1\right)}\right\Vert}_2^2\right)$ the noise is updated, and the standard deviation for several iterations *η* is a constant.

The difference between the proposed algorithm and PGPD [13, 25] is as follows: In this paper, the geodesic distance is used to replace the Euclidean distance in the PGPD algorithm to select the similar blocks and avoid the limitations of the Euclidean distance. Moreover, the proposed algorithm can search similar blocks in the whole image domain, while PGPD search for similar blocks in a slightly larger search window than the reference block. Here, the proposed algorithm is more accurate. PGPD adds no noise to image blocks for prior learning. In the proposed method, image blocks with additional Gaussian noise are used for prior learning. This technique gives full consideration to the noise affecting the image block sufficient information and to prior learning of additional noise image block directly. By using the property of the similar block group means value, the proposed method can reduce the noise in the image block, and the image block sparse representation can eliminate the noise signal; thus, the additional Gaussian noise in the image block can be eradicated.

## Prospects

Based on multi-layer neural network, deep learning with the feature of self-learning, which is input with massive data, has greater constructive and reasoning ability, thus, it can handle a variety of complex intelligent problems more effectively. In addition, deep learning also has more powerful learning ability and efficient feature expression ability Its more important advantage is that it can extract information layer by layer from pixel-level raw data to abstract semantic concept [26, 27], which makes it extracting the global features and context information of images more powerful and brings new ideas to solve traditional computer vision problems such as image segmentation and key point detection.

Therefore, it is believed that the application of deep learning in the 3-D reconstruction of cryo-EM images can exhibit better effects than the traditional methods, thereby it can enhance the resolution of biological macromolecules in 3-D reconstruction process. The next step is to use the parallel and deep learning method to realize the denoising algorithm put forward in the paper, which can reduce the time used to denoise the cryo-EM image.

## Abbreviations

2-D: Two-dimensional
3-D: Three-dimensional
BM3D: Block matching and three-dimensional filtering
CPV: Cytoplasmic polyhedrosis virus
Cryo-EM: Electron cryomicroscopy
CTF: Contrast transfer function
DCT: Discrete cosine transform
FPR: False positive rate
GMM: G
K-SVD: K-means singular value decomposition
MSE: Mean square error
NMR: Nuclear magnetic resonance
NSS: Nonlocal self-similarity
PDF: Probability density function
PGPD: Patch-group-based nonlocal self-similarity prior learning for image denoising
PSNR: Peak signal-to-noise ratio
SNR: Signal-to-noise ratio
SSIM: Structural similarity index measurement system
SURE: Stein’s unbiased risk estimator
TEM: Transmission electron microscopy
TNR: True negative rate
TPR: True positive rate
WGN: White Gaussian noise
